# External Electromagnet FPCB Micromirror for Large Angle Laser Scanning

**DOI:** 10.3390/mi10100667

**Published:** 2019-09-30

**Authors:** Karlmarx G. K. Periyasamy, Vixen Joshua Tan, Siyuan He, Nikolaos Kourtzanidis

**Affiliations:** Mechatronics and MEMS Research Laboratory, Ryerson University, Toronto, ON M5B 2K3, Canada; karlmarx.golanthan@ryerson.ca (K.G.K.P.); vjtan@ryerson.ca (V.J.T.); nkourtza@ryerson.ca (N.K.)

**Keywords:** flexible printed circuit board (FPCB) micromirror, external electromagnet, moving permanent magnet (PM), large aperture, high flatness

## Abstract

An external electromagnet plus moving PM (permanent magnet) FPCB (flexible printed circuit board) micromirror is proposed in this paper that can overcome two limitations associated with the previous FPCB micromirror with a configuration of an external PM plus moving coil, i.e., (1) it reduces the overall width beyond the mirror plate, and (2) increases the maximum rotation angle. The micromirror has two external electromagnets underneath an FPCB structure (two torsion beams and a middle seat) with two moving PM discs attached to the back and a metal-coated mirror plate bonded to the front of the FPCB middle seat. Modeling and simulation were introduced, and the prototype was fabricated and tested to verify the design. The achieved performance was better than that of the previous design: a maximum resonant rotation angle of 62° (optical) at a driving voltage of ±3 V with a frequency of 191 Hz, the required extra width beyond the mirror plate was 6 mm, and an aperture of 8 mm × 5.5 mm with a roughness of <10 nm and a flatness of >10 m (ROC, radius of curvature). The previous FPCB micromirror’s performance was: strain limited maximum rotation angle was 40° (optical), the extra width beyond the mirror plate was 14.7 mm, and had an aperture of 4 mm × 4 mm with a similar roughness and flatness.

## 1. Introduction

MEMS (microelectromechanical systems) micromirrors have been widely and successfully used in displays [1,2,3,4,5,6,7], optical switches [8], and medical devices [9] because of their small size, high integration, high reliability, and low cost. One major application of micromirrors is the laser scanner [10,11], such as for barcode reading [12], light detection and ranging (LiDAR) [13], depth sensing using structured light [14], bio-imaging [15,16,17,18,19], etc.

MEMS micromirrors can be classified into two categories according to the mirror plate‘s fabrication method and the aperture size. The first type has the mirror plate and actuator in the same body with an aperture of normally <1 mm [1,2,3,8,20] and surface flatness of ROC (radius of curvature) of normally <1 m. The second type can achieve a large aperture (several millimeters) with a high flatness (ROC >10 m), in which the mirror plate and actuator are fabricated separately and then bonded together afterwards [21,22]. For numerous laser scanning applications, such as laser engraving and LiDAR, a large aperture (e.g., one millimeter to several millimeters) and high flatness are preferred to accommodate a higher laser power or lower the laser power density, as well as achieve a better collimation. However, the second type needs to bond the mirror plate onto a released and fragile micro actuator, which is very difficult and then leads to a low bonding yield due to the low strain limit of the silicon flexible structure. In order to overcome this problem, flexible printed circuit board (FPCB) micromirrors were proposed and developed in [23,24,25,26] by the authors of the present paper, which have the mirror plate bonded to the FPCB structure. The flexible parts of FPCB structure are the polyimide layer or polyimide plus copper film laminate layer, which has a higher strain limit than silicon. Thus, the bonding operation becomes easier with a higher bonding yield. In addition, a larger rotation can be achieved due to the higher strain limit. Those FPCB micromirrors share the same advantages as the second type of MEMS micromirrors, i.e., a large aperture and high flatness. Two driving methods are used for those FPCB micromirrors, i.e., electrostatic [23,24,25] and magnetic with the configuration of “moving coil plus external permanent magnet (PM)” [26].

However there are two limitations associated with the previous FPCB magnetic micromirror, i.e., (1) a lot of extra space (width) beyond the mirror plate is required because of two external PMs located beside the FPCB structure [26], which are as large as 14.7 mm (width) × 6 mm (length along axial direction) beyond the mirror aperture of 4 mm × 4 mm; and (2) even though the maximum rotation angle is higher than that of the second type of traditional silicon MEMS micromirror, it can be further increased if the FPCB beams can be made of pure polyimide instead of polyimide plus copper laminate. This is because the copper film has a lower strain limit than that of polyimide. This paper proposes an FPCB micromirror to overcome these two limitations in the following two ways: (1) It utilizes the configuration of two moving PM discs plus two external electromagnets, which are underneath the FPCB structure. Thus, no external PMs beside the FPCB structure are needed, such as to reduce the overall extra width beyond the mirror plate. (2) No moving coils are needed to generate a Lorentz force. As a result, the torsion beams can be made of pure polyimide, which leads to a larger maximum rotation angle.

The advantages of the FPCB micromirror in this paper in comparison to a galvo scanner and rotation polygon mirror are: (1) lower cost since a mature and low-cost FPCB process and low-cost mirror plate fabrication (dicing metal coated wafer only) are used, and (2) it is not a motorized scanner, and so no bearing or lubrication is required; thus, it is more reliable.

Section 2 explains the design. Section 3 introduces the modeling and simulation. Section 4 shows the prototype and experimental test. Discussions and conclusions are summarized in Section 5.

## 2. Design

**Figure 1:**
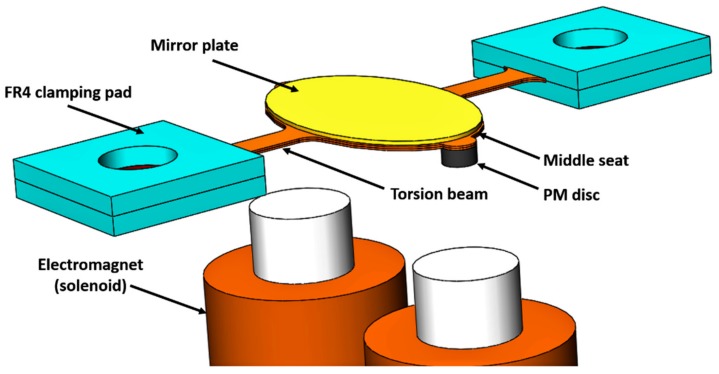
The external electromagnet flexible printed circuit board (FPCB) micromirror.

The external electromagnet FPCB micromirror consisted of the FPCB structure (elliptical middle seat with two small circular parts and two torsion beams), a mirror plate bonded to the middle seat and two PM (permanent magnet) discs attached to the two small circular parts, as shown in Figure 1. The two ends of the torsion beams were sandwiched by FR4 plates, which were screwed onto mechanical supporters (not shown in the Figure). The two small circular parts on the middle elliptical seat were only for convenience when bonding the PM disks, which are not necessary if the footprint needs to be further reduced. A thin copper film was on the middle seat, which did not contribute to rotation stiffness and was used for trying various adhesives. The current was applied to the electromagnets to generate the attractive (one side) and repulsive (the other side) forces between the electromagnets and PM discs to rotate the mirror.

Since the external electromagnets were located beneath the FPCB structure instead of beside it, the FPCB micromirror had a smaller footprint than the previous design [26] where the external PMs were located beside the FPCB structure. The FPCB micromirror in this paper could achieve a larger maximum rotation angle because the torsion beams were made of pure polyimide, which has a higher strain strength (by 2–3%) than the polyimide and copper laminate used in the previous design.

The FPCB torsion beams were composed of two polyimide layers (57 µm each). A copper layer was only on the middle seat where the mirror is bonded. The flexure structure was two torsion beams. The size of each torsion beam was 3 mm long and 1 mm wide with a thickness of 114 µm. The elliptical mirror plate was 8 mm × 5.5 mm × 0.12 mm. Each PM disc was 1 mm in diameter and 0.5 mm in thickness. Each solenoid was 19 mm in length and 6 mm in diameter with 1600 turns of copper wire. The distance between two solenoids’ centers was 8 mm.

## 3. Modeling and Simulation

### 3.1. Static Model

**Figure 2:**
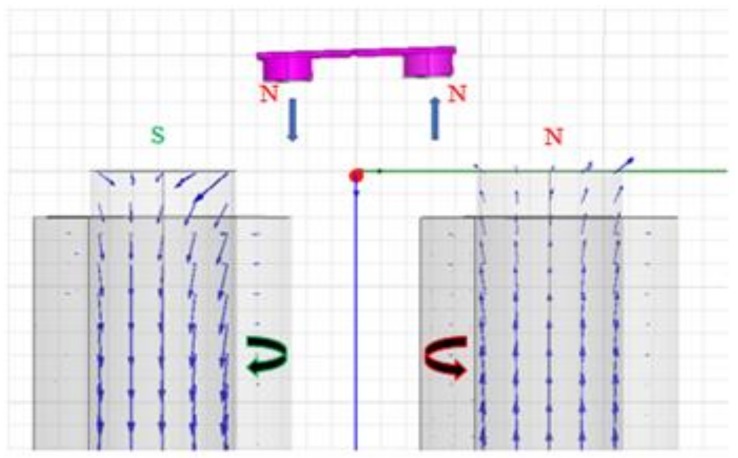
Simulation of torque *T*_e_ at the rotation angle *θ* and applied voltage *V*.

The relationship between the rotational angle and the voltage (or current) was modelled as follows. Equation (1) shows that the stiffness caused by mechanical torque is proportional to the mechanical rotation angle and stiffness:

*T*_m_ = *K θ* (1)

where *T*_m_ is the mechanical torque; *θ* is the mechanical rotational angle of the FPCB micromirror; and *K* is the rotational stiffness, which can be obtained via simulation using ANSYS Workbench [28]. First, for any rotation angle *θ* with any driving *V* voltages (current), the electromagnetic torque *T*_e_ generated between the two electromagnets (solenoids) and PM discs was obtained via simulation using the ANSYS Maxwell 3D module [29], as shown in Figure 2. Then the voltage V (current) was varied till *T*_e_ was equal to *T*_m_ at a rotation angle *θ*. Thus, the final voltage *V* corresponded to the rotation angle *θ*. Due to the static or very low driving frequency (<100 Hz), the impedance of the solenoid was considered fixed, i.e., only a resistance of 72.5 Ω was considered. Therefore, the voltage instead of the current was used for modeling and controlling since it was practically easier to adjust.

In simulations, the following parameters were used: (1) magnet disc: bulk electrical conductivity = 5.5 × 10^5^ S/m and magnetic coercivity = −9.0 × 10^5^ A/m; and (2) polyimide: Young’s modulus 2.5 GPa ~4 GPa [26], three values of modulus are used in simulations, i.e., 2.5 GPa, 3.2GPa [27] and 4 GPa. Poisson’s ratio = 0.34. The properties of the polyimide used here were from general data. However, the FPCB process affected the polyimide’s property. The simulation result is shown in Figure 5, which shows that the FPCB micromirror can achieve a ±3° optical rotation at ±5 V. A non-high static angle was pursued since the application in this paper was for the large angle laser scanning using resonant rotation.

### 3.2. Dynamic Simulation

**Figure 3:**
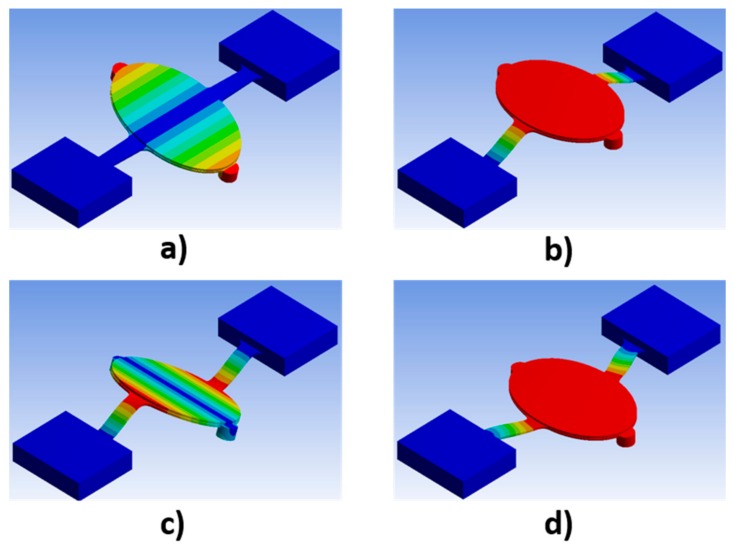
Resonant vibration modes of FPCB micromirror. (**a**) 1st mode; (**b**) 2nd mode; (**c**) 3rd mode; (**d**) 4th mode.

The resonant vibration modes were simulated using ANSYS workbench [28]. The results are shown in Figure 3. The first mode shown is rotation about torsion beams and the second mode is translation. Frequencies are: 207 Hz, 437 Hz, 1480 Hz and 3726 Hz when the modulus is 2.5 GPa; 235 Hz, 487 Hz, 1648 Hz and 4204 Hz when the modulus is 3.2 GPa; 261Hz, 546 Hz, 1847 Hz and 4699 Hz when the modulus is 4 GPa. Densities of the magnetic discs, the mirror plate, the copper film on elliptical middle seat, and polyimide were 7500 kg/m^3^, 2330 kg/m^3^, 8970 kg/m^3^, and 1420 kg/m^3^, respectively. The FPCB micromirror was designed to work at its first resonant rotation mode. In the future, the dimension of the torsion beams will be optimized to further increase the frequency difference between the first and second resonant vibration modes.

### 3.3. Stress Analysis

The von Mises stress in the FPCB structure was obtained using ANSYS static module. The yield strength (86–89 MPa) of FPCB polyimide [30] was used, which corresponded to the allowed maximum rotation close to 100° (optical) of the FPCB micromirror. For comparison, the design using the configuration of FPCB micromirror in Periyasamy et al. [26], i.e., moving coil plus external PM with copper film on torsion beams, was also simulated. In that design the allowed maximum rotation angle was limited to 40°(optical) because of the low strain limit of the copper film [31,32].

## 4. Prototype and Test 

### 4.1. Prototype

**Figure 4:**
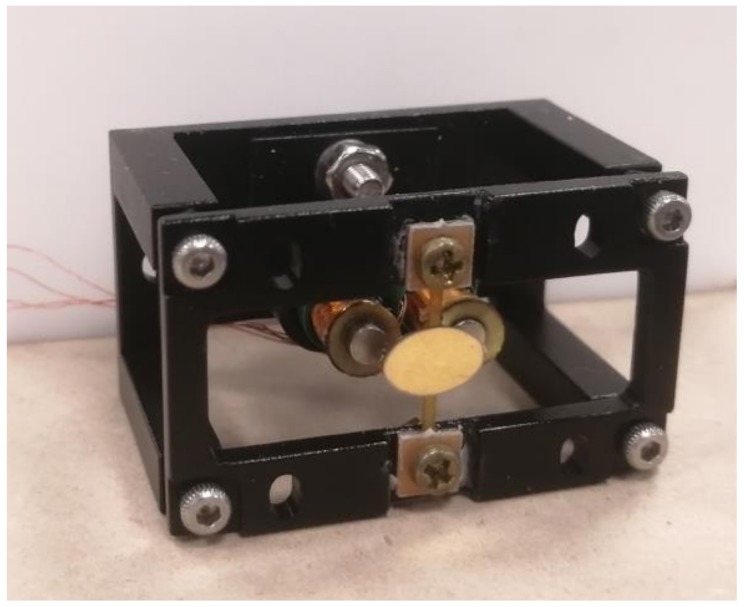
Prototype of the external electromagnet FPCB micromirror.

The FPCB structure and the gold-coated silicon mirror plate were fabricated independently and then bonded together using epoxy-based adhesive. The mirror plate was fabricated through the following two steps: (1) gold coating a silicon wafer with a thickness of 100 nm, and (2) Deep reactive-ion etching (RIE). The two magnetic discs were bonded to the circular parts of the FPCB middle seat, as shown in Figure 4. The aperture was 8 mm × 5.5 mm. The surface roughness was less than 10 nm and the flatness was ROC > 10 m (optical 3D profiler, Zygo microscope, Middlefield, CT, USA). The prototype was placed in a frame for the angular sensing using a photodiode, which is not presented in this paper. The extra space/width beyond the mirror plate was 6 mm, which was smaller than that of the previous design in Periyasamy et al. [26] (14.7 mm). Due to the manual assembly and the imperfection in the shape and dimension of solenoids, solenoids were not perfectly aligned with the PM discs; the distance between two solenoids > 8 mm and the gap could only be estimated to be 2.5–3 mm. Both were difficult to measure after assembly.

### 4.2. Static Test

**Figure 5:**
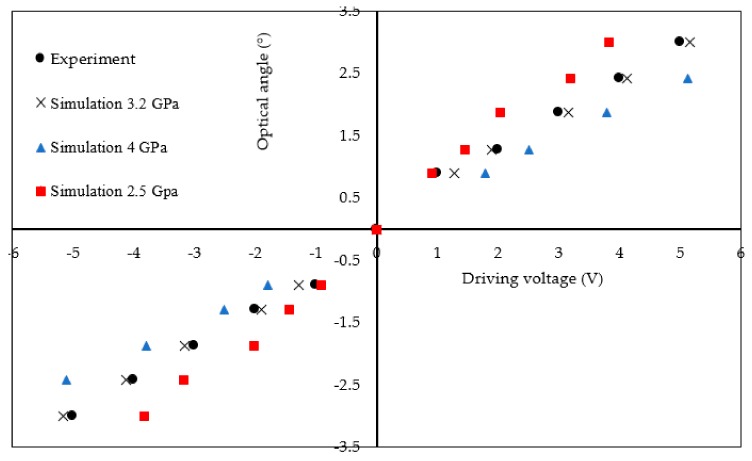
Simulated and tested static performance of the FPCB micromirror.

The static performance was obtained using a collimated laser (532 nm, <5 mW) and a grid paper. The optical rotation angle was from −3° to +3° at driving voltages of −5 V to 5 V. The results are plotted in Figure 5. Simulation results are also included for comparison. The discrepancy between the simulated and tested results could be attributed to two main reasons: (1) Inaccuracy in the gap between the PM discs and solenoids; any small assembly error caused an inaccuracy in the gap distance between the PM discs and the external solenoids leading to a discrepancy in the simulated and tested results. (2) Inaccuracy in polyimide’s properties (especially modulus) exist since only general FPCB polyimide properties before the FPCB process are currently available. The properties after the FPCB process are unavailable from the manufacturer. The FPCB process affects the polyimide properties. It seems the modulus of 3.2 GPa can achieve good match between simulation result and testes result. In addition, the polyimide was assumed to be isotropic in simulations for simplicity even though it is anisotropic. Due to the viscoelastic property of the polyimide, the prime method [26] was used to conduct the static test, e.g., a 1 Hz square wave was applied to the FPCB micromirror with an amplitude of ±1, ±2, ±3, ±4, or ±5 V.

In the simulations for this paper, the polyimide beams were simplified to be considered as elastic materials instead of viscoelastic materials. The viscoelasticity is to be studied in the future.

### 4.3. Dynamic Test

**Figure 6:**
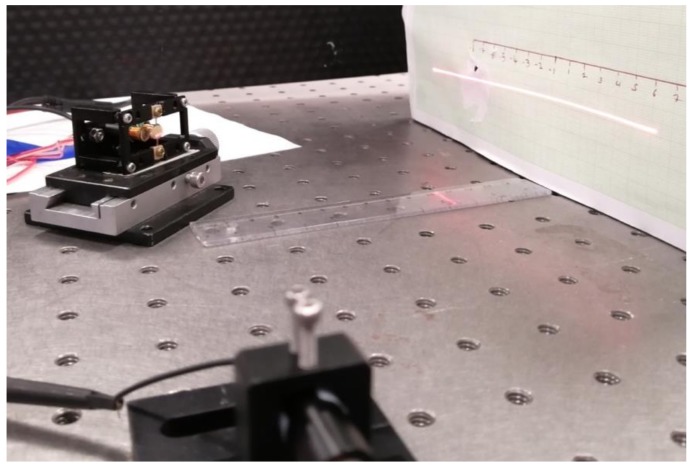
Resonant rotation of the FPCB micromirror for scanning laser.

**Figure 7:**
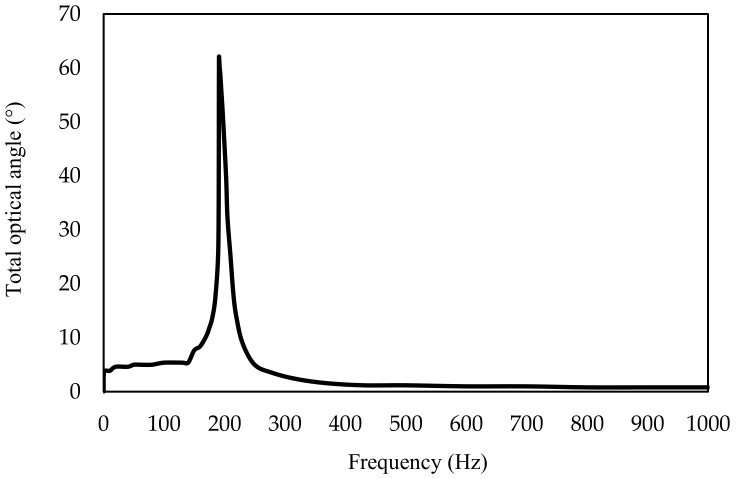
Tested results of dynamic rotation of the FPCB micromirror.

The dynamic rotation angle for the sinusoidal driving voltages of −3 V to 3 V over the frequency range of 0 Hz to 1000 Hz was tested, as shown in Figure 6 and Figure 7. The resonant frequency was 191 Hz with the optical rotation of 62°. The resonant frequency was lower than the simulation value. This could be contributed to the boundary condition, i.e., the simulation assumed exact clamping boundary condition while the prototype’s boundary condition could not be perfect clamped; the experimental tests revealed that when the prototype was well stretched, the resonant frequency could be 20–30 Hz higher and vice versa.

**Figure 8:**
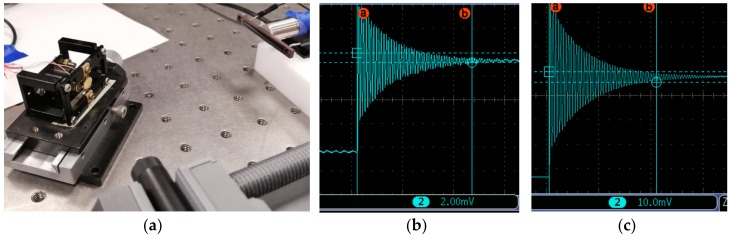
Step response test. (**a**) Step response test setup. (**b**) Step response curve from −1V to +1V. (**c**) Step response curve from −5V to +5 V.

When applying ±3 V, a resonant scanning of 62° (optical) was obtained, even though the maximum allowed close to 100°. To leave enough of a safety margin, less than 70° was practically realized in the prototype. However, the previous FPCB magnetic micromirror could only achieve an allowed maximum 40° optical scanning. The step response was obtained using a PSD (position sensing detector), as shown in Figure 8a. Figure 8b shows a step response when a step voltage of −1 V to +1 V (−0.89° to +0.89°) was applied. The settling time was 226 ms. Figure 8c shows a step response when a step voltage of −5 V to +5 V (−3° to +3°) was applied. The settling time was 206 ms. Oscillations within ±5% of the final value was used as the settled value. The translation mode should appear around 438 Hz. However, the testing setup cannot detect the translation. That is why there is no other peak beside the rotation peak till 1000 Hz in Figure 7.

### 4.4. Fatigue Test

**Figure 9:**
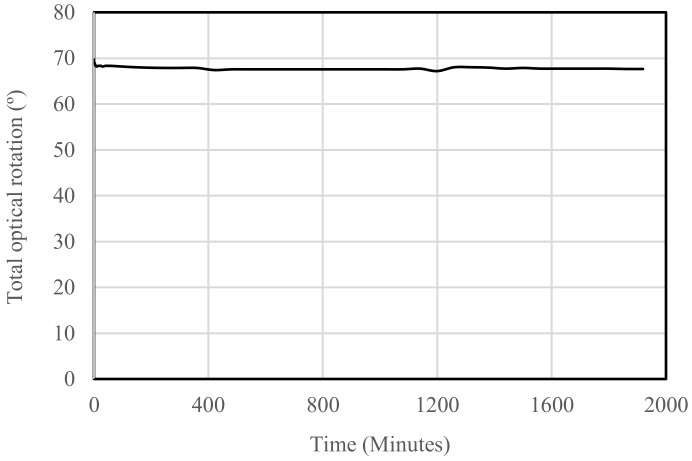
Fatigue test result.

At the time of writing, the FPCB micromirror is being tested for fatigue life. So far, results have shown that the resonant frequency becomes lower if the fatigue test is interrupted. A small fluctuation in rotation angle can be observed during the test. A continuous increase (like creep) has not been observed. This is probably because the fatigue test is done using completely inverse oscillations, i.e., ±34°. The fatigue test is on a table with various prototypes undergoing fatigue tests. The driving circuit is different from the one used for Figure 9.

## 5. Discussion

2D laser scanning can also be formed using two of the 1D FPCB micromirrors developed in this paper, where one is for vertical scanning (quasi-static oscillation) and the other is for horizontal scanning (resonant oscillation). More research is needed to study the viscoelastic property of the polyimide material. All simulations in this paper did not consider the viscosity property of the polyimide material and approximate the anisotropic polyimide material as isotropic material for simplicity. This approximation may lead to error, the quantification of which will be studied in the future. The simulation results only provide reference values. Other types of mesh will be tried in FEM simulation later instead of 3D mesh. In addition, the FPCB micromirror(s) will be integrated with single point LIDAR to construct a single-line or multiple-line scanning LiDAR (light detection and ranging).

Thus far, the alignment between the M discs and solenoids has been ensured by the FPCB micromirror holders and the solenoid holders. A misalignment does exist. The consequence is that there has been an undesired initial rotation or even translation, as well as unsymmetrical quasi-static rotation. However, no visible effect has been observed in the resonant oscillation, which was how the FPCB micromirror in this paper was used. In the future, a better solenoid holder and FPCB micromirror positioning mechanism will be developed. One disadvantage of the FPCB micromirror design in this paper is that the resonant frequency was reduced because the heavy PM discs were added to the moving FPCB middle seat. The high mass of PM discs causes high Q-factor and reduces the stability during the resonant scanning. In addition, the high mass also lowers the resonant frequency and makes the settling time long. However, the size and then the mass of PM discs do not need to be so high because there is still a lot of room to increase the current through the solenoids to increase the electromagnetic force even with smaller PM discs. The current PM disc is the smallest one we can find in the market. It can be smaller if customized design PM discs are used.

## 6. Conclusions

This paper developed an external electromagnet plus moving PM magnet FPCB micromirror which overcame the two limitations associated with the previous external PM magnet plus moving coil FPCB micromirror, i.e., (1) it reduced the footprint from 26 mm × 17.7 mm to 23 mm × 14 mm with an increased mirror aperture (4 mm × 4 mm to 8 mm × 5.5 mm), and (2) it increased the allowed maximum optical rotation angle from 40° to 62° (100° was the allowed maximum).
